# Cementing the Stepfamily? Biological and Stepparents’ Relationship Satisfaction After the Birth of a Common Child in Stepfamilies

**DOI:** 10.1177/0192513X19836456

**Published:** 2019-04-01

**Authors:** Katya Ivanova, Nicoletta Balbo

**Affiliations:** 1 University of Amsterdam, Amsterdam, Netherlands; 2 Bocconi University, Milan, Italy

**Keywords:** common child, fertility, relationship satisfaction, stepfamily, (step)parent

## Abstract

This article studies the relationship between having a common child in stepfamilies and partners’ relationship satisfaction. Previous works have primarily looked at children’s adjustment in stepfamilies and have cautioned against seeing a common offspring as a way to “cement” the partnership because the addition of a shared child does not benefit the child from an earlier union. We used seven waves of the German “Panel Analysis of Intimate Relationships and Family Dynamics” to examine the relationship satisfaction of partners in a stepfamily and its association with the potential birth of a common child. After controlling for initial relationship satisfaction, we see that having a common child is linked to higher satisfaction over time. Interestingly, for those whose common child is between 1 and 3 years old, we saw temporarily lower relationship satisfaction, which was less pronounced for the partner who was a stepparent in the context of the union.

Western countries have witnessed a growth in the instability of marriages and cohabitations over the past several decades, in combination with high rates of repartnering (Amato & James, 2010; Sweeney, 2010; Thomson, 2014). As the majority of relationship dissolutions involve couples with at least one minor child, this adult sequential monogamy has resulted in the proliferation of stepfamilies (unions of two adults with at least one child from an ex-partner). The presence of stepchildren, however, does not imply the discontinuation of childbearing. In fact, a number of studies have demonstrated that having children from a previous union does not suppress the transition to a new birth in the current partnership (e.g., Ivanova, Kalmijn, & Uunk, 2013; Jefferies, Berrington, & Diamond, 2000; Meggiolaro & Ongaro, 2010; Vikat, Thomson, & Hoem, 1999). As a result, we have witnessed an increase in the number of households where the partners have both common offspring as well as children from earlier partnerships.

A lot of the interest in these complex stepfamilies or blended families has focused on the adjustment and life outcomes of the children raised in the partnerships, with the somewhat consistent message that they fare worse than children raised in intact families (Coleman, Ganong, & Fine, 2000). One of the specific concerns has been how the addition of a half-sibling can affect the already present child of an earlier union, who is not biologically related to one of the partners. Some previous works have argued that the addition of a child does not benefit offspring from earlier unions and have, therefore, cautioned partners against seeing the addition of a new child as a way to “cement” the family (MacDonald & DeMaris, 1996; Stewart, 2005). What has remained underexplored, however, is the impact this fertility transition can have on the *partners’* assessment of the relationship. Studying how individuals’ assessment of their relationship might be affected by life transitions within repartnering unions is an essential question to address, given the reported higher volatility of these partnerships (Lyngstad & Jalovaara, 2010).

In our contribution, we focus explicitly on partners in stepfamilies. Existing research has examined individuals across types of family constellations; such examples are works on the adjustment of types of parents (e.g., a biological parent in an intact family vs. a stepparent in a stepfamily; Pace & Shafer, 2015) or on relationship satisfaction in different-order partnerships (e.g., Skinner, Bahr, Crane, & Call, 2002; Vemer, Coleman, Ganong, & Cooper, 1989). We argue that the study of the effect of life course transitions across types of families can lead to biased conclusions about the significance of these events, due to the potential existence of certain unobserved, difficult to measure characteristics that are related both to the likelihood of being involved in a reconstituted family and the outcomes of interest (relationship satisfaction in our case). Earlier works have indeed addressed those challenges by, for example, controlling in their models for observed individual characteristics (e.g., level of educational attainment, religiosity, and racial background; Pace & Shafer, 2015). In our contribution, we have chosen to focus explicitly on individuals residing in simple stepfamily households at the start of observation (i.e., where only the children of one of the partners are present in the household) and examine both partners’ relationship satisfaction over time and across the potential birth of a common child. In other words, we do not compare individuals across types of partnerships but, rather, examine individuals who have already self-selected into those more complex families at the start of observation.

In the theoretical section of our work, we begin by reflecting on the already documented link between the transition to parenthood and individual subjective well-being, as well as relationship satisfaction and marital quality. We then argue that having a common child in a stepfamily in particular might have additional benefits for the partners’ assessments of that union. We conclude that section by reflecting on whether these additional benefits might be more or less pronounced for one partner over the other, as a function of their stepparenthood status *within* the household.

## Theoretical Background

A lot of what we know about the interplay between fertility and marital quality stems from studies of intact families, where the focus has predominantly been on the transition to parenthood (i.e., birth of the first child) and individual subjective well-being (e.g., happiness, life satisfaction; Aassve, Arpino, & Balbo, 2016; Balbo, Billari, & Mills, 2013; Myrskylä & Margolis, 2014). The theoretical mechanisms proposed as underlying the link between fertility and life satisfaction in general focus on the financial, physical, and emotional costs associated with having children. The comparisons of parents and nonparents render mixed results with respect to who is better off in terms of individual well-being. However, when following parents over time, studies report that temporary changes in adjustment can be observed (with an increase in the year before the birth and a decrease in the first few postbirth years), with a general return to the prechild well-being levels in the longer run (e.g., Clark, Diener, Georgellis, & Lucas, 2008; Myrskylä & Margolis, 2014). What is also relevant for our study is the effect not just of the transition to parenthood but also of higher order births. Here, studies find either no changes in subjective well-being following the birth of a second child (e.g., Balbo & Arpino, 2016) or a negative effect (e.g., Kohler, Behrman, & Skytthe, 2005). Interestingly, when it comes to more explicit measures of relationship satisfaction and marital quality following the birth of a child, the findings more uniformly point to a somewhat stable drop (Dew & Wilcox, 2011; Doss, Rhoades, Stanley, & Markman, 2009; Keizer & Schenk, 2012; Nomaguchi & Milkie, 2003; for a meta-analysis, see Mitnick, Heyman, & Smith Slep, 2009).

As noted earlier, these studies have exclusively focused on intact partnerships. What we argue, however, is that fertility transitions in stepfamilies have an additional symbolic value, over and above satisfying the desire for children. One of the reasons proposed for why individuals have children is that a shared child can confirm a couple’s status as a “family” and signal the partners’ commitment to each other (the so-called commitment hypothesis; Griffith, Koo, & Suchindran, 1985; Vikat et al., 1999). This incentive could be even more pronounced within stepfamilies. It has been suggested that these families are less institutionalized, with rights and obligations in these unions being more ambiguous than in intact, first marriages (Cherlin, 1978). Therefore, having a common child in such a partnership could be even more important in reducing uncertainty about the status of the union and in cementing the partners’ commitment to the future of their relationship. In line with this argument, some previous works have demonstrated that the presence of children from an earlier partnership (i.e., already being a parent) does not necessarily suppress the transition to a birth in the current relationship, pointing to the symbolic significance of having a child with one’s partner (e.g., Anderson, 2000; Jefferies et al., 2000; Meggiolaro & Ongaro, 2010; Vikat et al., 1999). Given this line of reasoning, one can expect that having a common child in a stepfamily can have a positive effect on both partners’ assessment of the relationship, potentially due to the added symbolic significance of having that child within an otherwise less institutionalized family unit.

At this point, we need to also consider how the partners within a stepfamily might differ in how they experience fertility transitions, due to the presence of stepchildren for one of the parents. When considering the partner who is a stepparent within the partnership, we would expect an additional boost to their assessment of the relationship because the union is now better integrated through a blood tie. Prior to that common offspring, that partner could be seen as an “outsider” to the biological parent–child dyad. The birth of a mutual child, however, links everyone in the union and can make the stepparent feel like a legitimate member of the family group (Bernstein, 1989). At the same time, when we look at the partner who is the biological parent of all the children, the addition of a mutual child can in fact be linked to an additional increase in care burden. For example, previous studies have shown that the birth of a mutual child does not necessarily pull the stepparent into the care of the stepchild (MacDonald & DeMaris, 1996; Stewart, 2005). In other words, one parent could end up with a larger share of child care than the partner—both for the child from an earlier relationship as well as for the newborn common offspring. Stated differently, the addition of a shared child more closely resembles a higher parity transition for the biological parent than for the stepparent (at least insofar as coresident children are concerned), and such increases in the number of children have been linked to lower levels of relationship satisfaction (e.g., Elmslie & Tebaldi, 2014; Twenge, Campbell, & Foster, 2003).

In summary, in our work, we examine how having a common child within a stepfamily can affect the partners’ assessment of their intimate relationship. Though both partners are likely to benefit from the symbolic meaning of having that child within an otherwise less institutionalized family form (Cherlin, 1978), we expect to see a more pronounced gain in relationship satisfaction for the partner who was just a stepparent up to that point. In our work, we focus specifically on simple stepfamilies at the start of observation (i.e., households in which only the children from a previous union of one of the partners are present) to be able to clearly distinguish between the biological parent and stepparent statuses in the context of that household.

## Method

### Data and Analytical Sample

Our analyses are based on data from the first seven waves of the German “Panel Analysis of Intimate Relationships and Family Dynamics” (pairfam), Release 7.0, and its supplement DemoDiff (Brüderl et al., 2016). The German Family Panel pairfam started in 2008 with a sample of randomly selected persons (i.e., anchors) of three birth cohorts: 1991-1993, 1981-1983, and 1971-1973. DemoDiff is a survey of residents of eastern Germany that was designed to complement the German Family Panel (Kreyenfeld, Huinink, Trappe, & Walke, 2012). The final sample size at the first wave was 13,891 anchors. The data were collected annually, targeting both the main respondents as well as their potential partners. The sample sizes at each consecutive wave were *n* = 13,891 at Wave 1, *n* = 9,069 at Wave 2, *n* = 9,074 at Wave 3, *n* = 8,074 at Wave 4, *n* = 7,249 at Wave 5, *n* = 6,574 at Wave 6, and *n* = 5,919 at Wave 7. The largest drop of respondents was observed between the first and second waves, with attrition (defined as participants at *t* − 1 participating again at *t*) dropping substantially after that. Additional analyses have been carried out by Brüderl and colleagues (2018) comparing the realized pairfam sample at Wave 2 with other large, nationally representative German data collections, such as Socioeconomic Panel Study (Wagner et al., 2010). They found that, for example, the differences in income between pairfam, Wave 2 respondents, and Socioeconomic Panel Study 2010 participants were small (Brüderl et al., 2018.). In other words, it appears that the later waves of pairfam are comparable with other, nationally representative surveys in Germany, even after the initial loss of anchors. A detailed description of the study can be found in Huinink et al. (2011).

As we were interested in the association between childbearing and relationship satisfaction for biological and stepparents, we had rather specific data requirements for our analytical sample. First, we were interested in those anchors who reported having a partner at the time of the interview. Second, we needed only those unions that had residential biological children of only one of the partners at the time of the first observation (i.e., simple stepfamilies). Third, if the partners experienced the birth of a common child, it had to be reported as having happened after the start of the current partnership. Finally, we needed these partners to report on their own relationship satisfaction over multiple waves in order to examine the association between the potential birth of a common child and one’s relationship satisfaction. This led to a final analytical sample of 482 anchors, of which 452 reported on a single union and 30 reported on two unions (i.e., a final count of 512 unions). Table 1 displays the sample selection steps as well as the number of anchors who were left at each successive decision.^1^ In our analyses, we used the self-reported relationship satisfaction of the anchors and, if participating, their partners, accounting for the clustering of individuals in unions, as well as the fact that some unions were clustered in respondents (further explained in the Analytical Approach section).

**Table 1.** Analytical Sample Selection Steps.

	Original sample sizes	Step 1: Anchor reported having a partner at the time of the interview	Step 2: Step 1 + anchor reported resident noncommon children at first observation for the union	Step 3: Step 2 + excluding anchors whose first common child was born before the start of the union	Step 4: Step 3 + excluding anchors with only one observation for the union
DemoDiff	1,489 anchors	1,267 anchors	135 anchors	135 anchors	104 anchors
pairfam	12,402 anchors	9,585 anchors	617 anchors	616 anchors	378 anchors

### Measures

#### Relationship Satisfaction at the Final Observation

Our dependent variable was measured using the question “Overall, how satisfied are you with your relationship?” Answers were on a scale from 0 (*very dissatisfied*) to 10 (*very satisfied*). Both the anchors as well as their partners could indicate their relationship satisfaction at each wave of observation. Our outcome variable was measured at the final moment when that union was observed (which could be at any wave after the baseline observation of the partnership).

#### Stepparenthood Status

The main explanatory variable of interest was a dummy variable measuring whether the respondent is the step- or the biological parent of the resident noncommon children at the first observation of the union.

#### Transition to a Common Child

The other key explanatory variable in our analyses was whether the partners experienced the birth of a common child during the observation period (0 = *no birth* and 1 = *birth*). Importantly, for those who did experience the birth of a common child, we also accounted for the number of months that had passed since the birth at the time when the final relationship satisfaction was reported (i.e., at the final wave of observation for that union).

#### Control Variables

We controlled for several individual- and partnership-level characteristics. Foremost, we controlled for the gender (0 = *male*, 1 = *female*), educational level (low, middle, and high), and age in years of the participant whose relationship satisfaction we were analyzing. Additionally, we accounted for the age (in years) of the youngest noncommon resident child. Third, we controlled for the duration of the partnership (in months) at the end of the observation period (i.e., when the dependent variable was measured). Finally, we controlled for the relationship satisfaction of the respondent at the first observation for the partnership. Table 2 displays the descriptive statistics for our analytical sample.

**Table 2.** Descriptive Statistics of Analytical Sample (Partners Nested in 512 Unions), Separately for the Step- and Biological Parents.

	Bioparent		Stepparent		Total	
	Mean	*SD*	Mean	*SD*	Mean	*SD*
Relationship satisfaction at first observation	8.37	1.95	8.53	1.77	8.41	1.90
Relationship satisfaction at last observation	7.44	2.25	7.94	1.97	7.59	2.18
Experienced birth of a common (timing considered at last observation) child						
No birth					0.79	—
Birth was ≤12 months ago					0.06	—
Birth was >12 and ≤36 months ago					0.07	—
Birth was >36 months ago					0.08	—
Respondent is female	0.88	—	0.12	—	0.50	—
Age of respondent (years)	34.01	5.67	35.75	7.79	34.87	6.86
Educational level of respondent						
Primary education	0.25	—	0.28	—	0.27	—
Secondary education	0.50	—	0.43	—	0.46	—
Higher education	0.25	—	0.29	—	0.27	—
Duration of relationships at the end of observation (months)					65.30	53.44
Age of the youngest noncommon child					10.92	5.02

*Note. SD* = standard deviation.

### Analytical Approach

As stated above, our final analytical sample consisted of 512 unions, nested in 482 anchors, with some anchors reporting on two unions. We used the self-reported information on relationship satisfaction of both the anchor and the partner in that union (when available). This means that our data were composed of a single line per respondent, nested within unique partnerships. Each line contained the individual characteristics of the respondent (e.g., self-reported relationship satisfaction at the first and last observations) as well as the characteristics of the specific partnership (e.g., duration of the partnership at the final observation). We used random effect linear models at the couple level, with standard errors clustered in anchors (to account for the multiple unions reported by some), to estimate the association between the potential experience of the birth of a common child and the self-reported relationship satisfaction at the final observation (controlled for the initial level of satisfaction). Though the optimal analytical approach would have been to estimate couple-level fixed effects, where the change in relationship satisfaction of one parent is directly compared with that of the other parent, we decided against it for two main reasons. First, we did not have full information from both partners for each union, which would have resulted in prohibitively small cell counts. Second, we wanted to include in our analyses also stepfamilies that did not experience the birth of a common child during the period of observation.

In our analyses, we first examined whether the birth of a common child was associated with a boost in relationship satisfaction overall (Model 1). We then took into account how recent the potential birth of that common child was, by including a categorical variable where 0 = *no birth experienced*, 1 = *birth experienced within the previous year*, 2 = *birth experienced between 1 and 3 years ago*, and 3 = *birth experienced more than 3 years ago* (Model 2).^2^ We then estimated models that allowed us to test if the experience of a childbirth differed for the individuals who were a biological versus a stepparent in the context of the union (by including an interaction between the respondent’s parent type and the birth-of-a-common-child categorical variable; Model 3).

## Results

We begin by giving an impression of the relationship satisfaction of the partners at the first observation. As can be seen in Table 2, at the first observation, the self-reported relationship satisfaction of the partners was rather high (*M* = 8.41, *SD* (standard deviation) = 1.90 on a 0-10 scale), with no significant differences between the step- and biological parents. The difference does become statistically significant at the last moment the unions are observed, with the stepparents reporting somewhat higher relationship satisfaction, though the gap is not substantial (*M* = 7.44, *SD* = 2.25 for the biological parents and *M* = 7.94, *SD* = 1.97 for the stepparents, *t*(665) = 2.67, *p* < .05). In total, there were 107 births of a common child over the observation period for the stepfamilies in our analytical sample. As we were concerned about a potential high selectivity in our sample of stepfamilies with a common child compared with those without, we checked whether the partners who were happier at the first observation were more likely to have a child during the observation period. The difference in average self-reported relationship satisfaction between those who did and did not have a common child was statistically significant at *p* < .10 but substantively rather small (0.32 points higher for those who had a common child). More important, we also did not find a strong association between the self-reported relationship satisfaction at the first observation and the amount of time we observed the couples (i.e., *r* = .07, *p* < .10).

Table 3 displays the results from the estimated multivariable random effect regression models. Our first model focused purely on the association between having a child and relationship satisfaction. As can be seen in Model 1, there is a boost of more than 0.5 point in the final reported relationship satisfaction for those individuals who experienced the birth of a common child. Model 2 recognizes the potential time-specific association between the birth of a child and relationship satisfaction and shows the link documented in Model 1 for those who experienced the birth within the previous year, between 1 and 3 years ago, and more than 3 years ago. Interestingly, we see that the positive association between birth and satisfaction with the union is significant for those who experienced the birth either in the past year or more than 3 years ago (with both showing a boost of about 1 point in relationship satisfaction). The individuals whose common child was a toddler, however, reported somewhat lower relationship satisfaction than those who did not experience the birth of a shared offspring (though the difference was not statistically or substantially significant).

**Table 3.** Random Effect Models at the Couple Level to Estimate the Effect on Relationship Satisfaction of Being a Biological Parent or a Stepparent at Different Points in Time After the Birth of a Common Child.

	Coefficient (*SE*)		
	Model 1	Model 2	Model 3
Relationship satisfaction at the first observation	0.365** (0.063)	0.365** (0.063)	0.368** (0.064)
The participant is a stepparent	0.275 (0.194)	0.270 (0.194)	0.130 (0.229)
Birth of a common child is observed	0.578** (0.219)		
Birth of a common child (reference = none)			
≤12 months ago		0.868* (0.361)	0.787* (0.395)
>12 and ≤36 months ago		−0.150 (0.400)	−0.449 (0.484)
>36 months ago		0.950** (0.274)	0.981** (0.281)
Interaction between birth of common child and participant parent status			
Stepparent × ≤12 months ago			0.727 (0.543)
Stepparent × >12 and ≤36 months ago			1.244* (0.501)
Stepparent × >36 months ago			−0.032 (0.602)
Participant is female	−0.064 (0.193)	−0.091 (0.193)	−0.096 (0.193)
Educational level of respondent (reference = middle)			
Low	0.221 (0.235)	0.196 (0.233)	0.197 (0.232)
High	−0.003 (0.200)	−0.023 (0.199)	−0.021 (0.199)
Age of participant at the first observation (years)	0.017 (0.019)	0.017 (0.019)	0.020 (0.019)
Duration of union at the final observation (months)	−0.000 (0.002)	−0.001 (0.002)	−0.000 (0.002)
Age of youngest noncommon child at the first observation (years)	−0.002 (0.023)	−0.004 (0.022)	−0.006 (0.023)
Constant	3.700** (0.876)	3.792** (0.882)	3.680** (0.892)

*Note. SE* = standard error.

* *p* < .05. **p < .01. ***p < .001.

**Figure 1.**
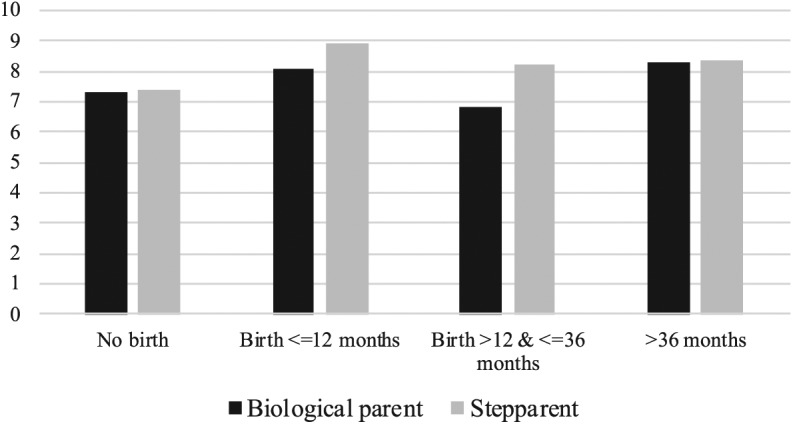
Estimated margins for self-reported relationship satisfaction, by parent type and experience of common childbirth (Model 3, Table 3). *Note.* Margins are presented at representative values (all categorical variables at reference category and continuous variables at the mean).

The final step of our analyses was to examine whether the birth of a common child is experienced in a similar fashion by the partner who was a stepparent and the one who was a biological parent at the start of observation. In other words, we examined if the addition of a common child was potentially more “beneficial” for one type of parent over the other. The question is addressed in Model 3 of Table 3 (with the marginal effects for the two types of partners plotted in Figure 1). What we can see in that model is that differences between step- and biological parents can only be observed in the period with a temporary dip in relationship satisfaction (i.e., between 1 and 3 years after the birth of a child). This difference is substantially meaningful, with the stepparent reporting 1.37 points higher relationship satisfaction in that period than the partner. In other words, what we see in that final model is that when a drop in relationship satisfaction is reported, it is more pronounced for the person who is adding *another* biological child to the household. This is in line with previous findings showing that higher parity fertility transitions are linked to lower levels of relationship satisfaction (e.g., Elmslie & Tebaldi, 2014; Twenge et al., 2003).

## Discussion

The proliferation of stepfamilies in the past few decades has been associated with an increasing scientific interest in these partnerships. A lot of the academic debate thus far has centered on the potential higher volatility of these unions (Lyngstad & Jalovaara, 2010) and on the repercussions for the adjustment of the children raised within these families (Coleman et al., 2000). What has remained somewhat on the sidelines, however, is the study of the partners’ own experience of their union, especially in light of any potential further fertility transitions within the partnership. Though theoretically the birth of a common child can be seen as a way to cement the family status of the union, earlier work has cautioned against viewing common children as a way to solidify stepfamilies due to concerns about the outcomes for the child who is not biologically related to both parents (e.g., Stewart, 2005). We built on this line of work by shifting the focus from concern about the children raised in stepfamilies to considering the potential benefits of having a common child for the partners involved in that household. We addressed two research questions: first, whether the birth of a common child is positively associated with the partners’ relationship satisfaction and, second, whether that potential link differs according to the partners’ stepparenthood status at the start of the union. Our findings point to a positive association between the birth of a common child and individual relationship satisfaction, with some interesting short-term differences in the evaluations of the step- and the biological parent.

Our first research question was grounded in the theoretical mechanisms postulated by the literature on fertility transitions in higher order unions and, in particular, the so-called commitment hypothesis (Griffith et al., 1985; Vikat et al., 1999). According to that mechanism, the partners in any family composition benefit from the birth of a common child, partly because the transition confers the status of a “real” family. Given the lower institutionalization of stepfamilies (Cherlin, 1978), we suggested that the partners involved in these partnerships might have even more to gain from such a transition. Indeed, our findings demonstrated that even after controlling for initial relationship satisfaction levels and the duration of the partnership, those who experienced the birth of a common child reported more than a 0.5-point boost to their final assessment of the union compared with their counterparts without a common child.

Our second research question focused on whether or not the biological parent and the stepparent in a partnership experience the birth of a common child differently. We expected that because of the added benefit of having a common child for the stepparent (i.e., by providing a biological link for that partner to the biological parent–child dyad), we might see a more pronounced increase to that partner’s satisfaction. Our results showed that the more substantial differences between the partners were not found in the overall boost to relationship satisfaction but, rather, during the period when a temporary dip in relational satisfaction was observed. It was at this moment that we saw that the biological parent of all the children assessed the union less favorably than the partner. To better understand this result, future investigations should also consider how the caring load is divided between the partners following the transition to having a common child. It is possible that what we were observing in our analyses was the result of a potentially higher caring load for the individuals who were biological parents of all offspring (i.e., acting as primary caregiver for their own child from a previous union plus the added care for the recently born common child). This explanation is in line with earlier works on the transition to parenthood and relationship satisfaction in non-reconstituted families, which have reported similar dips in the assessment of the relationship following the birth of a child and have mostly pointed to potential misbalances in the division of household labor as the driving mechanism (e.g., Keizer & Schenk, 2012). Such an analysis of the division of care between the partners in a stepfamily with a common child is beyond the scope of our work, particularly given the sample restrictions, but is an important issue to investigate in future studies.

The findings of our work need to be considered in light of several important caveats. Foremost, though we put forward potential theoretical mechanisms that might explain our findings, we are unable to test these explicitly. A significant step forward in this line of enquiry will be a dynamic investigation of the division of labor within the union to better understand the emerging differences between the step- and biological parents. Another challenge in this work, as with many other studies of stepfamilies, is the lower number of stepmothers compared with stepfathers. Frequently, the study of stepfamilies is in fact an investigation of stepfathers and biological mothers. All of our models include a control variable for the gender of the reporting partner. However, we cannot disregard the possibility that our findings might be affected by the fact that stepfathers and biological mothers are overrepresented in our analytical sample. It is also important to note here that we have not accounted for the presence of biological children outside the household. In other words, it is possible that some of the individuals designated as “stepparents” were biological parents to nonresident children. Our current theoretical arguments are largely household focused, reflecting on what the impact of an additional child *within* the household might be for the partners. However, future works should examine how stepparents experience the birth of a common child depending on whether they already have biological children of their own outside the household.

An important point that needs to be recognized is the selective attrition in our sample. In other words, it could be the individuals who were less satisfied with their relationship whom we observed for a very short time and for whom we did not observe the birth of a child. Similarly, when considering the time since the experienced birth, we might have been left with only the happier participants at each successive interval. We addressed this shortcoming, to the best of our abilities given the sample restrictions, by controlling for “baseline” relationship satisfaction in all the models. We also checked whether the individuals who were less satisfied at the first observation were present for a shorter period of time in our analytical sample but the association between first reported union satisfaction and the number of months observed was very weak. Yet we acknowledge that we cannot definitively conclude that our findings are causal—in other words, that having a common child *leads* to higher relationship satisfaction. Further studies can follow up on our findings by applying more rigorous methodological approaches when larger, high-quality longitudinal analytical samples of stepfamilies become available. For example, person–fixed effect models would allow for biological parents’ or stepparents’ relationship satisfaction to be modeled over time, with the person serving as his or her own reference point.

Despite the methodological shortcomings of our work, we would like to leave the reader with two main take-home messages. First, though the study of stepfamilies has often focused on the children raised in these households, we argue that it is also important to consider how the partners involved in these families experience their relationship and possible family-related life transitions. The tentative conclusion of our work is that such a “concrete baby” (Ganong & Coleman, 1994) might indeed benefit the adults involved. Yet what we also see in our work is that these gains might be unequally distributed between the partners. As revealed in studies focusing on the initial transition to parenthood, the unbalanced division of labor following the birth could have important repercussions for the parents involved.

## Notes

1. It is important to note here that the final step in the sample selection (Step 4) meant that we were potentially selecting those unions where the transition to having a common child was more likely. Additional analyses showed that relationship satisfaction at the first observation was indeed higher in our analytical sample than among the excluded couples. The excluded couples were also together for a shorter period of time and had a younger nonshared child in the household. In other words, when considering the findings of our analyses, it is important to keep in mind that, overall, we were studying the association between childbirth and relationship satisfaction in potentially more stable and satisfying partnerships.

2. The delineation of these periods was driven by findings of earlier works, as well as being a reflection of the German public child care system. Previous studies have shown a clear drop in well-being in the first year following the birth of a child, with adjustment remaining somewhat stable thereafter (e.g., Myrskylä & Margolis, 2014). Additionally, what is important to note about the German context is that though children are entitled to a place in public child care facilities after the age of 1 year, almost universal enrollment in child care is observed only after the age of 3 years, with rates being substantially lower until that age (Organisation for Economic Co-operation and Development, 2018). Therefore, given our arguments about the care burden associated with having an additional child, we chose to divide the periods as outlined above.

## References

[bibr1-0192513X19836456] Aassve A. Arpino B. Balbo N. (2016). It takes two to tango: Couples’ happiness and childbearing. European Journal of Population, 32, 339-354. doi:10.1007/s10680-016-9385-1 30976218 PMC6240998

[bibr2-0192513X19836456] Amato P. R. James S. (2010). Divorce in Europe and the United States: Commonalities and differences across nations. Family Science, 1, 2-13.

[bibr3-0192513X19836456] Anderson K. G. (2000). The life histories of American stepfathers in evolutionary perspective. Human Nature, 11, 307-333. doi:10.1007/s12110-000-1006-2 26193656

[bibr4-0192513X19836456] Balbo N. Arpino B. (2016). The role of family orientations in shaping the effect of fertility on subjective well-being: A propensity score matching approach. Demography, 53, 955-978. doi:10.1007/s13524-016-0480-z 27306764

[bibr5-0192513X19836456] Balbo N. Billari F. C. Mills M. (2013). Fertility in advanced societies: A review of research. European Journal of Population/Revue Européenne de Démographie, 29, 1-38. doi:10.1007/s10680-012-9277-y 23440941 PMC3576563

[bibr6-0192513X19836456] Bernstein A. C. (1989). Yours, mine, and ours: How families change when remarried parents have a child together. New York, NY: Macmillan.

[bibr7-0192513X19836456] Brüderl J. Hank K. Huinink J. Nauck B. Neyer F. J. Walper S.. . . Wilhelm B. (2016). The German Family Panel (pairfam) (GESIS Data Archive, ZA5678 data file Version 7.0.0). Cologne, Germany: GESIS. doi:10.4232/pairfam.5678.7.0.0

[bibr8-0192513X19836456] Brüderl J. Hank K. Huinink J. Nauck B. Neyer F. J. Walper S.. . . Wilhelm B. (2018). The German Family Panel (pairfam) (GESIS Data Archive, ZA5678 data file Version 9.1.0). Cologne, Germany: GESIS. doi:10.4232/pairfam.5678.9.1.0

[bibr9-0192513X19836456] Cherlin A. (1978). Remarriage as an incomplete institution. American Journal of Sociology, 84, 634-650. doi:10.1300/J087v26n01_10

[bibr10-0192513X19836456] Clark A. E. Diener E. Georgellis Y. Lucas R. E. (2008). Lags and leads in life satisfaction: A test of the baseline hypothesis. Economic Journal, 118, F222-F243.

[bibr11-0192513X19836456] Coleman M. Ganong L. Fine M. (2000). Reinvestigating remarriage: Another decade of progress. Journal of Marriage and the Family, 62, 1288-1307. doi:10.1111/j.1741-3737.2000.01288.x

[bibr12-0192513X19836456] Dew J. Wilcox W. B. (2011). If momma ain’t happy: Explaining declines in marital satisfaction among new mothers. Journal of Marriage and Family, 73, 1-12. doi:10.1111/j.l741-3737.2010.00782.x

[bibr13-0192513X19836456] Doss B. Rhoades G. Stanley S. Markman H. (2009). The effect of the transition to parenthood on relationship quality: An 8-year prospective study. Journal of Personality and Social Psychology, 96, 601-619. doi:10.1037/a0013969 19254107 PMC2702669

[bibr14-0192513X19836456] Elmslie B. T. Tebaldi E. (2014). The determinants of marital happiness. Applied Economics, 46, 3452-3462. doi:10.1080/00036846.2014.932047

[bibr15-0192513X19836456] Ganong L. Coleman M. (1994). Remarried family relationships. Thousand Oaks, CA: Sage.

[bibr16-0192513X19836456] Griffith J. D. Koo H. P. Suchindran C. M. (1985). Childbearing and family in remarriage. Demography, 22, 73-88. 3979617

[bibr17-0192513X19836456] Huinink J. Brüderl J. Nauck B. Walper S. Castiglioni L. Feldhaus M. (2011). Panel Analysis of Intimate Relationships and Family Dynamics (pairfam): Conceptual framework and design. Zeitschrift Für Familienforschung, 23, 77-100.

[bibr18-0192513X19836456] Ivanova K. Kalmijn M. Uunk W. (2013). Fertility after repartnering in the Netherlands: Parenthood or commitment? Advances in Life Course Research, 21, 101-112. 26047545 10.1016/j.alcr.2013.08.003

[bibr19-0192513X19836456] Jefferies J. Berrington A. Diamond I. (2000). Childbearing following marital dissolution in Britain. European Journal of Population, 16, 193-210. doi:10.1023/A:1026529300659

[bibr20-0192513X19836456] Keizer R. Schenk N. (2012). Becoming a parent and relationship satisfaction: A longitudinal dyadic perspective. Journal of Marriage and Family, 74, 759-773. doi:10.1111/j.1741-3737.2012.00991.x

[bibr21-0192513X19836456] Kohler H. P. Behrman J. R. Skytthe J. R. (2005). Partner + children = happiness? The effects of partnerships and fertility on well-being. Population and Development Review, 31, 407-445.

[bibr22-0192513X19836456] Kreyenfeld M. Huinink J. Trappe H. Walke R. (2012). DemoDiff: A dataset for the study of family change in eastern (and western) Germany. Schmollers Jahrbuch, 132, 653-660.

[bibr23-0192513X19836456] Lyngstad T. H. Jalovaara M. (2010). A review of the antecedents of union dissolution. Demographic Research, 23, 257-291.

[bibr24-0192513X19836456] MacDonald W. L. DeMaris A. (1996). Parenting stepchildren and biological children: The effects of stepparent’s gender and new biological children. Journal of Family Issues, 17(1), 5-25. doi:10.1177/019251396017001002

[bibr25-0192513X19836456] Meggiolaro S. Ongaro F. (2010). The implications of marital instability for a woman’s fertility: Empirical evidence from Italy. Demographic Research, 23, 963-996. doi:10.4054/DemRes.2010.23.34

[bibr26-0192513X19836456] Mitnick D. M. Heyman R. E. Smith Slep A. M. (2009). Changes in relationship satisfaction across the transition to parenthood: A meta-analysis. Journal of Family Psychology, 23, 848-852. doi:10.1037/a0017004 20001143 PMC2812012

[bibr27-0192513X19836456] Myrskylä M. Margolis R. (2014). Happiness: Before and after the kids. Demograhy, 51, 1843-1866. 10.1007/s13524-014-0321-x 25143019

[bibr28-0192513X19836456] Nomaguchi K. M. Milkie M. A. (2003). Costs and rewards of children: The effects of becoming a parent on adults’ lives. Journal of Marriage and Family, 65, 356-374.

[bibr29-0192513X19836456] Organisation for Economic Co-operation and Development. (2018). Enrolment in childcare and pre-school. Retrieved from https://www.oecd.org/els/soc/PF3_2_Enrolment_childcare_preschool.pdf

[bibr30-0192513X19836456] Pace G. T. Shafer K. (2015). Parenting and depression: Differences across parental roles. Journal of Family Issues, 36(11), 1001-1021. doi:10.1177/0192513X13506705

[bibr31-0192513X19836456] Skinner K. B. Bahr S. J. Crane D. R. Call V. R. A. (2002). Cohabitation, marriage, and remarriage: A comparison of relationship quality over time. Journal of Family Issues, 23, 74-90. doi:10.1177/0192513X02023001004

[bibr32-0192513X19836456] Stewart S. D. (2005). How the birth of a child affects involvement with stepchildren. Journal of Marriage and Family, 67, 461-473.

[bibr33-0192513X19836456] Sweeney M. M. (2010). Remarriage and stepfamilies: Strategic sites for family scholarship in the 21st century. Journal of Marriage and Family, 72, 667-684. doi:10.1111/j.1741-3737.2010.00724.x

[bibr34-0192513X19836456] Thomson E. (2014). Family complexity in Europe. Annals of the American Academy of Political and Social Science, 654, 245-258. doi:10.1177/0002716214531384

[bibr35-0192513X19836456] Twenge J. M. Campbell W. K. Foster C. A. (2003). Parenthood and marital satisfaction: A meta-analytic review. Journal of Marriage and Family, 65, 574-583. doi:10.1111/j.1741-3737.2003.00574.x

[bibr36-0192513X19836456] Vemer E. Coleman M. Ganong L. H. Cooper H. (1989). Marital satisfaction in remarriage: A meta-analysis. Journal of Marriage and the Family, 51, 713-725.

[bibr37-0192513X19836456] Vikat A. Thomson E. Hoem J. M. (1999). Stepfamily fertility in contemporary Sweden: The impact of childbearing before the current union. Population Studies, 53, 211-225.

[bibr38-0192513X19836456] Wagner G. G. Frick J. R. Schupp J. Anger S. Giesselmann M. Goebel J.. . . Spieß K. C. (2010). German Socioeconomic Panel Study (SOEP), data of the years 1984-2010 (data file Version 27). doi:10.5684/soep.v27

